# Ketone bodies: from enemy to friend and guardian angel

**DOI:** 10.1186/s12916-021-02185-0

**Published:** 2021-12-09

**Authors:** Hubert Kolb, Kerstin Kempf, Martin Röhling, Martina Lenzen-Schulte, Nanette C. Schloot, Stephan Martin

**Affiliations:** 1 Faculty of Medicine, University of Duesseldorf, Moorenstr. 5, 40225 Duesseldorf, Germany; 2 West-German Centre of Diabetes and Health, Duesseldorf Catholic Hospital Group, Hohensandweg 37, 40591 Duesseldorf, Germany; 3 Redaktion Deutsches Ärzteblatt, Reinhardtstr. 34, 10117 Berlin, Germany

## Abstract

**Abstract:**

During starvation, fasting, or a diet containing little digestible carbohydrates, the circulating insulin levels are decreased. This promotes lipolysis, and the breakdown of fat becomes the major source of energy. The hepatic energy metabolism is regulated so that under these circumstances, ketone bodies are generated from β-oxidation of fatty acids and secreted as ancillary fuel, in addition to gluconeogenesis. Increased plasma levels of ketone bodies thus indicate a dietary shortage of carbohydrates. Ketone bodies not only serve as fuel but also promote resistance to oxidative and inflammatory stress, and there is a decrease in anabolic insulin-dependent energy expenditure. It has been suggested that the beneficial non-metabolic actions of ketone bodies on organ functions are mediated by them acting as a ligand to specific cellular targets. We propose here a major role of a different pathway initiated by the induction of oxidative stress in the mitochondria during increased ketolysis. Oxidative stress induced by ketone body metabolism is beneficial in the long term because it initiates an adaptive (hormetic) response characterized by the activation of the master regulators of cell-protective mechanism, nuclear factor erythroid 2-related factor 2 (Nrf2), sirtuins, and AMP-activated kinase. This results in resolving oxidative stress, by the upregulation of anti-oxidative and anti-inflammatory activities, improved mitochondrial function and growth, DNA repair, and autophagy. In the heart, the adaptive response to enhanced ketolysis improves resistance to damage after ischemic insults or to cardiotoxic actions of doxorubicin. Sodium-dependent glucose co-transporter 2 (SGLT2) inhibitors may also exert their cardioprotective action via increasing ketone body levels and ketolysis. We conclude that the increased synthesis and use of ketone bodies as ancillary fuel during periods of deficient food supply and low insulin levels causes oxidative stress in the mitochondria and that the latter initiates a protective (hormetic) response which allows cells to cope with increased oxidative stress and lower energy availability.

**Keywords:**

Ketogenic diet, Ketone bodies, Beta hydroxybutyrate, Insulin, Obesity, Type 2 diabetes, Inflammation, Oxidative stress, Cardiovascular disease, SGLT2, Hormesis

## Background

In recent reviews, we have described the role of elevated endogenous insulin levels in the development of obesity and arteriosclerosis [1, 2]. The pathophysiological mechanisms of hyperinsulinemia are numerous as increased tubular sodium reabsorption or unfavorable effects on lipid metabolism. Reduction of endogenous insulin secretion leads to increased breakdown of fatty acids which will be discussed in this review.

Fat has turned out to be a superior form of energy reserve because more energy can be stored per weight and volume, and no additional water is required for maintaining solubility and conformation. Glycogen has a glucose to water ratio of 1:2 (weight/weight) and therefore contains about 7 times less calories per weight than fat. In the human body, glycogen stored in the muscle is primarily consumed locally during muscle work. Glucose stored as liver glycogen (80–100 g in adults) may be released into the bloodstream, but this is insufficient to maintain normal body functions for more than a day but may last for a few days because of concurrent gluconeogenesis. By contrast, energy stored as fat in adipose tissue and ectopic sites may provide energy for weeks. There are no protein stores; muscle fibers serve as the primary source of amino acids for energy production [3].

The lack of food therefore leads to the preferential breakdown of fat, with a major contribution of increased lipolysis because of lowered insulin levels. When most of the glucose in glycogen stores is used up, digestion of fatty acids in the liver is upregulated, but under these circumstances, energy metabolism hardly can proceed beyond the generation of acetyl-CoA. This is due to the limited availability of oxaloacetate for oxidation in the tricarboxylic acid (TCA) cycle in hepatocytes because of its consumption during concurrent gluconeogenesis. Accumulation of acetyl-CoA is resolved by its conversion to acetoacetate, the majority of which is reduced to d-β-hydroxybutyrate (βOHB); another part spontaneously decarboxylates to acetone. These three “ketone bodies” are released into circulation and taken up by other tissues, including the brain and heart, as an alternative source of energy [4–6]. An increase of the systemic level of ketone bodies thus indicates conditions of limited food supply, or at least limited dietary carbohydrate availability. We therefore discuss here that ketone bodies not only substitute for glucose as an external source of energy but also support the body in adapting to periods of limited food supply.

## Main text

### Metabolic conditions causing ketosis

Ketosis is caused by the preferential breakdown of fats for energy production, resulting in ketone body formation in the absence of sufficient carbohydrate (sugars or starches, glycogen) availability leading to low systemic insulin levels.

Reasons for ketosis include the following:

- Energy production preferentially from body fat reserves because of insufficient energy supply from dietary sources including digestible carbohydrates less than 5–10% of daily required energy (voluntary or during famine). The resulting decrease of insulin levels to the low normal range allows enhanced lipolysis. Such a situation may also occur during prolonged exercise. A normal blood glucose level is maintained by hepatic gluconeogenesis.
- Energy production preferentially from dietary rather than body fat but not from dietary carbohydrates because of very low digestible carbohydrates (daily max. 25–50 g for adults, “ketogenic diet”) in diets which are not restricted for the content of fat or protein (ketogenic diet, very low carbohydrate diet, paleolithic diet, low insulin diet). A normal blood glucose level is maintained by hepatic gluconeogenesis.
- Persons with diabetes type 1 (rarely type 2): Preferential energy production from dietary or body fat but not from dietary carbohydrates, because of insufficient ability to use blood glucose as an energy source because of too low insulin levels or very high insulin resistance such as during infections. A normal blood glucose level cannot be maintained because of the influx of glucose from dietary sources and/or the liver but decreased insulin-dependent efflux from the blood and lymph into tissues, resulting in hyperglycemia. In non-diabetic persons, endogenous glucose production from the liver usually is suppressed by the postmeal rise of blood insulin levels.

### Generation of ketone bodies and their metabolism

**Fig. 1:**
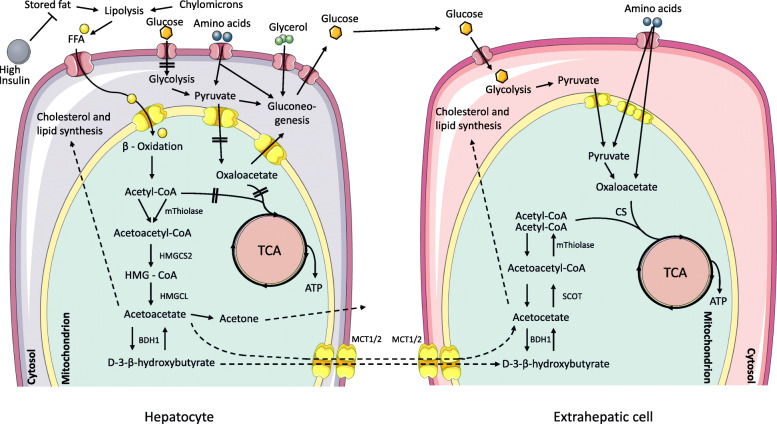
Overview of the ketogenesis and ketolysis pathways. In cases of limited availability of oxaloacetate, beta oxidation of fatty acids in hepatocytes leads to the accumulation of acetyl-CoA which is channeled into the ketogenic pathway and converted to acetoacetate, the majority of which is reduced to βOHB, another part spontaneously decarboxylates to acetone. Secreted βOHB and acetoacetate are taken up by extrahepatic cells and converted back to acetyl-CoA. The latter can be entered into the TCA cycle after conjugation with oxaloacetate by citrate synthase because there is no gluconeogenesis that would drain local pools of pyruvate and oxaloacetate. FFA, free fatty acids; mThiolase, mitochondrial thiolase; HMGCS2, hydroxy methylglutaryl-CoA synthase; HMGCL, HMG-CoA lyase; BDH1, mitochondrial βOHB dehydrogenase; MCT1/2, monocarboxylate transporter 1 and 2; SCOT, succinyl-CoA:3-oxoacid-CoA transferase; CS, citrate synthase

Energy production from fat requires the release of free fatty acids from triglycerides deposited in body fat stores or from dietary fat in chylomicrons or other triglyceride-rich lipoproteins [7, 8]. The release of fatty acids from adipocytes requires low insulin activity because this anabolic hormone is a potent inhibitor of lipolysis [1]. Free fatty acids in the plasma are taken up by most cell types for energy production, but primarily, hepatocytes can use them to generate ketone bodies for distribution as an alternative fuel to other cell types of the body if there is a lack of diet-derived glucose (Fig. 1).

Breakdown of fatty acids occurs in the mitochondria by beta oxidation yielding ATP and acetyl-CoA. In the absence of sufficient glycolysis-derived oxaloacetate plus the consumption of oxaloacetate for gluconeogenesis, and because of additional not well-researched metabolic factors, hepatocytes channel only little acetyl-CoA into the TCA cycle for terminal oxidation and further ATP production [6]. Rather, acetyl-CoA accumulates and is converted to acetoacetate via acetoacetyl-CoA (by mitochondrial acetoacetyl-CoA thiolase) and hydroxy methylglutaryl-CoA (by HMG-CoA synthase) from which acetoacetate and acetyl-CoA are cleaved (by HMG-CoA lyase). A small proportion of acetoacetate spontaneously decarboxylates to acetone and CO_2_, and a larger part is reduced to βOHB (by mitochondrial βOHB dehydrogenase, BDH1). The three “ketone bodies” (acetoacetate, βOHB, acetone) are secreted to the venous circulation, followed by uptake in extrahepatic tissues, excretion in the kidney, or exhaling (acetone) via the lungs. Acetoacetate may also be used in the cytosol for cholesterol synthesis and possibly lipogenesis [6, 9] (Fig. 1). The term ketone body usually includes βOHB, although its keto group is reduced to a hydroxyl group.

Significant amounts of ketone bodies are also produced during a non-ketogenic diet, leading to blood levels of around 0.05 mmol/l for acetoacetate, 0.05–0.4 mmol/l for βOHB, and 0.02–0.05 mmol/l for acetone. The levels vary over the day, decreasing with the uptake of carbohydrates and increasing in periods between meals or because of prolonged muscle work. After 2 weeks of fasting, levels of acetoacetate may reach 1 mmol/l and that of βOHB 5 mmol/l. Acetone concentrations in the blood may increase to 0.5 mmol/l [10–12]. A small fraction of ketone bodies reach the urine and can be reabsorbed to some degree, proportionate to circulating concentrations.

The uptake of acetoacetate and βOHB into the mitochondria of extrahepatic tissues follows a concentration gradient via monocarboxylate transporters 1 and 2. The use of ketone bodies for energy production is limited to extrahepatic tissues and is initiated by the oxidation of βOHB back to acetoacetate by mitochondrial BDH1. Acetoacetate is in equilibrium with acetoacetate-CoA via succinyl-CoA:3-oxoacid-CoA transferase (SCOT), and acetoacetate-CoA is cleaved by mitochondrial acetoacetyl-CoA thiolase yielding two molecules of acetyl-CoA. The latter are conjugated with oxalacetate (from glucose or amino acid catabolism) by citrate synthase to enter the TCA cycle or may be used for lipid synthesis [6] (Fig. 1). Hepatocytes cannot use ketone bodies for energy production because of the lack of SCOT which prevents ketolysis and futile cycling of acetoacetate back to HMG-CoA.

### Role of ketone bodies in energy homeostasis

Ketogenesis and the use of ketone bodies for energy production occur in most extrahepatic tissues including the brain (excluding erythrocytes and most malignant cell types). There is a major advantage from an evolutionary point of view because the ability to survive periods of starvation is substantially augmented. In the absence of ketogenesis, brain cells would entirely depend on hepatic and renal gluconeogenesis during long-term starvation. Substrates for glucose synthesis are limited in the body; they include glucogenic amino acids, glycerol from triglycerides, recycled lactate, and pyruvate via the Cori cycle (and ketone bodies). It has been calculated that the brain of an adult person might survive 2–3 weeks from gluconeogenesis alone but remains functional for at least 2 months if ketone bodies derived from fat depots are being used as an additional energy source. An obese person could even withstand a much longer period of starvation. After several weeks of fasting, two-thirds of the energy needed by the brain are provided by βOHB and acetoacetate [10]. The human brain also requires ketone bodies during the early postnatal phase. The metabolism of newborns is ketotic due to the low lactose content of colostrum. Nearly half of the energy consumed by the newborn human brain is from βOHB. After a few days of lactation, the lactose content has increased, and ketosis disappears [10, 13].

Another organ critical for survival is the heart. Interestingly, myocardial cells make little use of glucose for energy production but strongly rely on the oxidation of fatty acids which accounts for 60–85% of ATP produced. Additional energy substrates include glucose/lactate, ketone bodies, and amino acids [14, 15]. The use of acetoacetate and βOHB is proportionate to systemic levels so that there is increased consumption of ketone bodies during ketosis although free fatty acids remain the major substrate for ATP production [6].

The contribution of acetoacetate and βOHB to ATP production in the skeletal muscle varies substantially. After an overnight fast, ketone bodies contribute 10–20% to energy provision which may rise to 50% after several days of starvation. More than half of the energy comes from blood glucose [15, 16]. The disposal of ketone bodies to the skeletal muscle during aerobic exercise may rise up to fivefold, followed by post-exercise ketosis (0.3–2.0 mmol/l) depending on nutritional status and exercise intensity [17].

Taken together, the liver continuously produces low levels of ketone bodies during lipid catabolism, with a rapid increase in response to decreased availability of diet-derived glucose/pyruvate for channeling breakdown products of fatty acids into the TCA cycle for complete oxidation (Table 1). The ability of acetoacetate and βOHB to substibute for blood glucose in energy production is essential for survival during prolonged starvation, in particular, in regard to brain function.

**Table 1:** Ranges of ketone body levels in human plasma in different physiological conditions

Physiological condition	β-Hydroxybutyrate concentration in plasma (range in mmol/l)
Normal circadian variation	0.1–0.4
After prolonged exercise	0.3–2
After 1–2 days of fasting	1–2
After 2–3 weeks of fasting	5–7
After 1–3 weeks of ketogenic diet	0.5–5
During diabetic ketoacidosis	3–25

### Ketone bodies as a guardian angel

There may be a broader role of ketogenic diets in protecting body functions than simply causing less production of insulin and providing ketone bodies as alternative ancillary fuel. A rise of ketone body levels in the blood is indicative of fat breakdown in the absence of sufficient carbohydrate availability and the resulting low insulin secretion, for instance, during food shortage. Therefore, an increase of the systemic levels of acetoacetate or βOHB could be used by the body as a danger signal indicating risk of starvation followed by an appropriate response to modulate physiological mechanisms of relevance for improving survival during starvation. This chapter will discuss such a scenario.

During food shortage or starvation, the increased energy production from ketone bodies is associated with enhanced radical oxygen species (ROS) release in the mitochondria, a concomitant decrease of NADH in favor of NAD^+^ levels and a lower AMP/ATP ratio [18–21]. The enhanced production of ROS is also seen when exposing rat hepatocytes or human endothelial cells to acetoacetate [22, 23]. Markers of oxidative stress were also induced in bovine hepatocytes by βOHB or acetoacetate [24, 25]. In addition to increased ROS production from the mitochondria, NADPH oxidase 4 is activated in human endothelial cells by high concentrations of acetoacetate (4 mmol/l) and βOHB (12 mmol/l) [26]. Oxidative stress usually is accompanied by or leads to the activation of inflammatory reactivity and to cell damage at the level of lipids, proteins, and DNA. Indeed, βOHB was found to induce the pro-inflammatory cytokines tumor necrosis factor-α (TNFα) or interleukin (IL)-1β and IL-6 as well as the chemokine CCL2 in human microvascular endothelial cells or calf hepatocytes [24, 27].

**Fig. 2:**
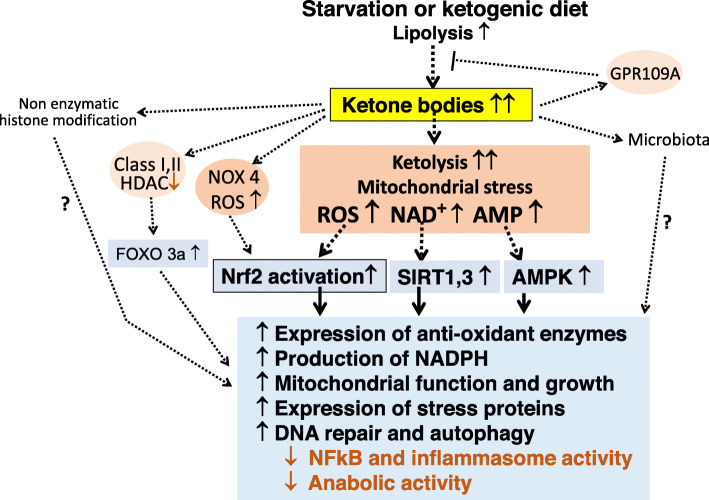
Scheme of cell-protective functions of ketone bodies. The metabolic shift towards fat oxidation and ketolysis during starvation or ketogenic diet is associated with mitochondrial stress characterized by increased levels of ROS and increased ratios of NAD^+^/NADH and AMP/ATP as well as AMP/ADP. These “danger signals” cause a protective adaptive (hormetic) cellular response, via the activation of Nrf2, SIRT1, SIRT3, and AMPK, respectively. Ketone bodies also activate ROS production from NOX4, and βOHB alters the gene expression pattern by promoting histone acetylation via inhibiting class I and II HDACs and possibly by direct β-hydroxybutyrylation of histones. Long-term consequences of the initial moderate metabolic stress include upregulation of anti-oxidative and anti-inflammatory activities and improved mitochondrial function. ROS, radical oxygen species; Nrf2, nuclear factor erythroid 2-related factor 2; SIRT, sirtuin, silent information regulator; AMPK, AMP-activated kinase; NFkB, nuclear factor kappa B; NOX, NADPH oxidase; HDAC, histone deacetylase; FOXO, forkhead box O

In view of such undesired consequences for cell physiology, it seems counterintuitive to consider ketosis and ketone bodies as beneficial to the organism. However, the above findings are contrasted by a large number of reports which link feeding a ketogenic diet or exposure to ketone bodies to the upregulation of anti-oxidant and anti-inflammatory mechanisms (reviewed in [6, 9, 28]). These seemingly controversial findings are resolved when considering that there is a time axis in the response to ketogenic diets or exogenous ketone bodies. The initial rise of ROS and pro-inflammatory mediators is followed by an adaptive cellular defense response which leads to prolonged upregulation of cell-protective activities including increased anti-oxidative and anti-inflammatory activity, cell repair, and regeneration mechanisms. These cellular responses are mediated by several danger-responsive regulatory molecules including the nuclear factor erythroid 2-related factor 2 (Nrf2), histone deacetylases of the sirtuin (SIRT) family, and AMP-activated kinases (Fig. 2).

The experimental evidence that ketone bodies need to first impair mitochondrial function before causing a beneficial response is mostly derived from animal models or cell culture. In rats, a ketogenic diet was found to enhance the production of H_2_O_2_ from the brain mitochondria accompanied by a decrease of glutathione levels in the liver. However, in subsequent weeks, hydrogen peroxide levels decreased below control. This adaptive response was carried by the accumulation of Nrf2 in the cell nuclei leading to the production of Nrf2-responsive targets such as NAD(P)H:quinone oxidoreductase and heme oxygenase 1 [29]. In a rat model of spinal cord injury, ketogenic diet induced the activation of Nrf2 which led to the attenuation of inflammation by decreasing the expression of nuclear factor “kappa-light-chain-enhancer” of activated B cells (NF-kB) expression and of pro-inflammatory cytokines IL-1β, TNFα, and interferon-γ (IFNγ) as well as by enhancing superoxide dismutase and decreasing myeloperoxidase activity [30]. The decreased activation of the NOD-, LRR- and pyrin domain-containing protein 3 (NLRP3) inflammasome in human monocytes or mice after treatment with βOHB [31] may also be the result of suppressed NF-kB activity. Pretreatment of human endothelial cells with ketone bodies for 48 h caused an adaptive response which prevented the oxidative damage seen after subsequent exposure to 4 mmol/l βOHB plus 1 mmol/l acetoacetate in controls. The protective effect of pretreatment with ketone bodies apparently was mediated by activation of Nrf2 [32]. Feeding rats a ketogenic diet or exogenous βOHB rendered the retina resistant to ischemic degeneration. Treatment with βOHB did not add to the protective effects of the ketogenic diet suggesting that cell protection by ketogenic diet was mediated by diet-induced ketone bodies. The diet as well as βOHB supplementation caused activation and nuclear translocation of Nrf2. Its causal role in rendering retina cells resistant to ischemic stress was proven by the loss of such protective effect when the Nrf2 gene was inactivated [33]. Feeding a calorie-restricted diet to mice also caused activation of Nrf2 and the expression of anti-oxidant enzymes; no such response was seen in mice with a disrupted Nrf2 gene [34]. Interestingly, the lifespan-extending property of a calorie-restricted diet in *Caenorhabditis elegans* was also lost in the absence of a functioning Nrf2//SKiNhead (SKN)-1 gene [35].

Taken together, ketone bodies appear to initially induce the production of excess ROS from the mitochondria which causes the induction of Nrf2, the master regulator of several hundred genes involved in cell protection, repair, and regeneration including DNA repair, autophagy, decreased endoplasmic reticulum stress, and improved mitochondrial function and growth but otherwise reduced anabolic activities to protect energy reserves [36–38]. Several pathways may lead to Nrf2 activation, i.e., translocation to the nucleus and binding to anti-oxidant/electrophile response elements in the promoter region for enhanced gene expression [39–41]. Of these, the major pathway involves the interaction of ROS with lysine residues of KEAP which blocks its ability to bind and deliver newly synthesized Nrf2 to the proteasomal degradation route (Fig. 2).

Since starvation is a life-threatening situation, it is not surprising that βOHB probably employs more than one pathway of dealing with the consequences of food shortage. Closely associated with the production of increased ROS from mitochondria leading to the activation of Nrf2 is a decrease in the NAD^+^/NADH ratio which causes the activation of NAD-dependent histone decacetylase sirtuins 1 and 3. These enzymes promote the enhanced expression of a set of genes also involved in anti-oxidant and anti-inflammatory activities as well as supporting autophagy, mitochondrial function, and growth, partially overlapping with the gene set activated by Nrf2 [42, 43]. A further important activity of sirtuin 3 is the deacetylation and activation of the NADP-dependent isocitrate dehydrogenases in the mitochondria and cytoplasm leading to increased NADPH production for efficient neutralization of lipid peroxides [9]. Evidence for the induction of sirtuin 3 by βOHB comes from studies of forebrain neurons [44]. In vivo, supplementing βOHB increased sirtuin 3 and mitochondrial respiration in the hippocampus of mice [45]. Concomitantly, there was reduced oxidative stress and decreased infarct volume after ischemic stroke [46]. Sirtuin 1 enzyme activity was found to be increased in murine hippocampal neurons when incubated with βOHB which also caused improved mitochondrial respiration [47].

The third sensor of energy homeostasis is the AMP/ATP (and AMP/ADP) ratio. Cells respond to a lower ATP level with upregulation of AMP-activated kinase(s) which promote cell-protective activities which overlap with those induced by Nrf2 or sirtuins, including the support of mitochondrial function and growth as well as regulating anti-oxidative, anti-inflammatory, and cell repair functions (DNA repair, autophagy), in part via the forkhead box protein O (FOXO) 3α transcription factor pathway [43, 48, 49]. For instance, the increase of βOHB levels leads to AMPK activation in vitro and in rats [50] as well as in mice [51] or cows [52].

We conclude that during nutritional ketosis, acetoacetate and/or βOHB cause an oxidative and metabolic stress situation that induces signaling via Nrf2 and sirtuins 1 and 3 and activation of AMPK resulting in a protective adaptive response characterized by improved mitochondrial function, an anti-oxidative and anti-inflammatory state, decreased endoplasmic reticulum stress, reduced anabolic metabolism, and strengthened cell repair mechanisms such as DNA repair and autophagy (Fig. 2). The downregulation of anabolic metabolism is also seen when feeding ketone esters in the context of an unrestricted diet, as shown by a decrease in blood glucose and insulin levels [53, 54]. The phenomenon that mild/moderate oxidative or otherwise damaging stress causes an adaptive response conferring stress resistance has been first observed in toxicological studies and has been named hormesis [55]. Studies during recent years have observed hormesis to underly a large number of physiological responses. For instance, hormesis appears to be essential for the beneficial effect of exercise, which initially causes oxidative and pro-inflammatory stress locally, and at the systemic levels followed by an adaptive protective response involving the total organism. Neutralization of exercise-induced ROS by anti-oxidative supplements prevented the upregulation of Nrf2 and training effects such as better muscle performance and mitochondrial function [56, 57]. Similarly, the health effects of many plant polyphenols depend on the electrophilic stress caused by the phytochemicals, upregulation of Nrf2 followed by anti-oxidative, and anti-inflammatory gene expression. Where analyzed, inactivation of the Nrf2 gene prevented the beneficial response to dietary phytochemicals [1, 58, 59].

For the induction of an oxidative stress response, βOHB may bypass the hormesis route and directly promote the gene expression of the anti-oxidant protein metallothionein and of the transcription regulator FOXO3a which mediates many protective actions of sirtuins. This is achieved by binding to class I and II histone deacetylases leading to enhanced acetylation nuclear histones including the promoter region of FOXO3a and metallothionein, in vitro and in several organs of mice, at βOHB concentration typical of nutritional ketosis [6, 60]. These findings have recently been challenged in a study of several mammalian cell types. Strong inhibition of histone deacetylases was not observed for βOHB but only for butyrate [27]. It was suggested that nonenzymatic beta-hydroxybutyrylation of lysine sites of histones may contribute to the anti-oxidant effect of ketone bodies [61]. Studies comparing the hormesis route of an adaptive cell response to βOHB exposure with that of direct modifying histone acetylation have not been performed; thus, the contribution of the latter to beneficial effects of nutritional ketosis remains unclear.

As a short-chain fatty acid molecule, βOHB is expected to possibly bind to several proteins with appropriate lipid binding sites, in addition to histone deacetylation inhibitors. Some experimental data are available for G protein-coupled receptors (GPRs). βOHB was found to antagonize binding/signaling of the agonist propionate of GPR41, resulting in decreased activation of sympathetic ganglia and sympathetic tonus in mice [62]. However, near maximal inhibition was already seen at 0.1 mmol/l βOHB which corresponds to physiological levels seen in the absence of nutritional ketosis.

Another target of βOHB is GPR109A (hydroxycarboxylic acid receptor 2). Here, the half-maximal effective concentration is around 0.8 mmol/l [63]. Therefore, GPR109A-dependent effects are expected to occur during ketosis. The closely related receptor GPR109B is activated by an intermediate product of fat beta oxidation, 3-OH-octanoid acid, also at concentrations related to ketosis [64]. Both receptors mitigate lipolysis from adipocytes and hence represent a counterregulatory loop for preventing excessive fatty acid breakdown and concomitant ketoacidosis (Fig. 2).

A further potential target of ketone bodies is the microbiota. This topic is not yet well researched. A recent meta-analysis [65] identified three trials studying the possible impact of carbohydrate-restricted diets on the microbiome of persons with obesity. The results concur in a decrease of Firmicutes bacteria with concomitant less butyrate production [66–68]. Whether this is a direct effect of ketone bodies, a consequence of the probably lower amounts of dietary fiber or of the concomitant weight loss remains to be elucidated.

### Clinical experience with ketogenic diets

The results of controlled clinical trials of fasting or very-low-calorie ketogenic diets of 1–3 weeks duration concur in a beneficial impact on body physiology. The expected loss of body weight is characterized by a preferential decrease of abdominal and ectopic fat stores such as in the liver or pancreas. Fasting glucose and insulin levels typically are lowered by 20% or more. Serum markers of oxidative stress, such as malonedialdehyde, and oxidative damage to cellular components are reduced. Systemic low-grade inflammation, such as a mildly increased level of C-reactive protein, is ameliorated, and hypertension is mitigated. Non-randomized trials of mild nutritional ketosis in persons with type 2 diabetes also report lower C-reactive protein concentrations in the intervention group [69–71]. Another consequence of fasting or very-low-calorie diets is lowering of blood levels of total cholesterol together with LDL cholesterol and triglycerides [72–74].

In all but one trial, body weight loss was substantially higher after several weeks or months in the groups with a calorie-restricted ketogenic diet compared with standard calorie-restricted non-ketogenic diets as control. In some trials, it was not tried to keep daily calorie uptake exactly identical between the groups. It may also be important that the macronutrient mass intake is similar between the groups [75]. Participants were overweight or obese, some trials included persons with type 2 diabetes [76–85]. During a dietary intervention period of 1–12 months, loss of body weight was 2–4 times higher in the low-calorie ketogenic diet group compared with the low-calorie non-ketogenic diet group (body weight loss by 4.4–23.7% or 5.8–27 kg versus 0.3–8.3% or 0.3–9.0 kg). Where analyzed, loss of total body fat and/or the decrease in waist circumference was also more pronounced in the group on ketogenic calorie-restricted diet compared with calorie restriction alone [73].

Ketogenic diets without calorie restriction (< 5–10% energy from carbohydrates) also have beneficial physiological responses. In the 1920s, ketogenic diets have been introduced to treat epilepsy in children, in particular, in drug-resistant epilepsy. A recent systematic review concluded that children on a ketogenic diet compared to usual care have an about 3 times higher chance of seizure freedom and an about 6 times higher chance of seizure reduction ≥ 50% of baseline. A parallel meta-analysis of trials in infants and adults did not find a significant effect [86, 87]. Currently, it is not known whether the mechanism responsible for preventing seizures involves the switch from glucose to ketone body utilization as an energy source or the modulation of neuronal signaling pathways by ketone bodies [88]. Beneficial effects of ketogenic diets appear to include a significant reduction of clinical symptoms of further brain disorders such as Alzheimer’s or Parkinson’s disease, anxiety, depression, or alcohol withdrawal symptoms [89–92].

To date, the largest experience with non-calorie-restricted ketogenic diet probably is in persons with obesity, metabolic syndrome, or type 2 diabetes. Because of the very low content of digestible carbohydrates in this type of diet, it has a very low glycemic index. There are very low postprandial rises of glucose levels which induce only a little insulin production. The lack of hyperinsulinemia is accompanied by a decrease in insulin resistance, and the latter usually is associated with metabolic improvement, weight loss, and lower blood pressure [93]. When compared with a non-calorie-restricted conventional or low-fat diet, the strongest loss of body weight is observed in the group of carbohydrate-restricted ketogenic diet in overweight/obese persons [94–101]. A recent meta-analysis of obese participants with type 2 diabetes reported a stronger decrease of HbA1c compared with the control diet (difference 0.5%). Levels of fasting insulin and of insulin resistance (HOMA-IR) decreased more strongly during the ketogenic diet [102]. The difference in triglyceride and HDL cholesterol levels was also significantly in favor of the ketogenic diets whereas LDL cholesterol levels increased in response to ketogenic diets. All metabolic effects of ketogenic diets were similar in obese nondiabetic study participants, but of lower magnitude [102]. The increased circulating concentrations of LDL cholesterol during a ketogenic diet may not be detrimental because there is a change in the composition of LDL subclasses, favoring large-sized buoyant LDL with cardioprotective properties over atherogenic smaller dense particles [103–107]. An increase of LDL cholesterol concentrations in the blood was not seen when most of the saturated fats in the diet (75%) were replaced by polyunsaturated fat [108]. A meta-analysis of randomized-controlled diet trials reported that replacing carbohydrates for saturated fat did not impact liver fat content (summary of 12 trials), but there was a reduction of liver fat if unsaturated fat was used in comparison with saturated fat (3 trials) [109]. Because of the clinically relevant effects on glycemic control, the American Diabetes Association has endorsed low-carbohydrate diet as part of medical nutrition therapy options in diabetic patients in 2019 [110].

Our research of published randomized controlled trials did not allow a safe estimate of the metabolic benefit of ketogenic diets in absolute terms. Many trials compared a non-calorie-restricted ketogenic diet with a hypocaloric (low fat) diet and therefore had to be excluded. The remaining trials focused on measures of body weight and fat mass, as described above. Fasting blood glucose was determined in six trials and was lower compared with the comparator diet by 0.7, 0.2, or 10 mmol/l, respectively [94, 97, 98, 100, 101, 106]. Levels of fasting insulin decreased more strongly with the ketogenic diet in 2 of 4 trials, by 1.1 or 3.6 μU/ml, respectively [97, 100]. In the other two trials, fasting insulin levels also were lower with the ketogenic diet, but differences did not reach the level of significance, possibly because of the low number of participants and because there was an unexpected decrease of insulin levels also with the comparator diets [94, 106].

As pointed out above, feeding a ketogenic diet is associated with a decrease in insulin secretion because of the low amounts of digestible carbohydrates. There is an association between the decrease of fasting insulin levels and loss of body weight, also in non-ketogenic diets [97, 111]. We have argued previously that insulin is the key driver of weight gain (or weight loss) because of the regulation of lipogenesis versus lipolysis by insulin which is seen already with hormone concentrations in the high normal versus low normal range [1]. Low postprandial insulin levels during a ketogenic diet therefore may be a critical factor accounting for the observed loss of body weight and fat.

Ketogenic diets are being tried in many other chronic disease conditions, such as inflammatory/autoimmune diseases, cancer, or polycystic ovary syndrome, and are being tested for improving physical performance in athletes or for promoting healthy aging. The results of further research have to be awaited.

### (Pre)clinical experience with supplementation of ketone bodies

Probably, the largest experience with supplementing ketone bodies comes from patients with a failing heart or appropriate animal models. Supplementing ketone bodies in the absence of a ketogenic diet creates an “artificial” metabolic situation because increased levels of ketone bodies are otherwise only seen if the energy metabolism relies on fat breakdown because of little dietary glucose. One approach of supplementing ketone bodies is the use of calcium and sodium salts of a racemic mixture of βOHB which resulted in a modest increase of circulating levels, to about 0.5 mmol/l. There were gastrointestinal problems and possible long-term risks because of high sodium intake [112]. Much more effective are βOHB esters such as (R)-3-hydroxybutyl (R)-3-hydroxybutyrate which can be given per os to reach > 4 mmol/l of βOHB and was well tolerated during a 28-day trial, except for bitter taste [113, 114].

Ketone bodies have become of interest in the context of heart diseases because fat and ketone body breakdown is the predominant pathway in the myocardium for energy production, including the not-ketotic state, i.e., in the presence of normal glucose supply. A recent quantitative analysis of the arteriovenous gradient for metabolites observed no net extraction of glucose by the non-failing human heart. It was calculated that about 85% of cardiac ATP production was from free or lipoprotein-derived fatty acids, 6.4% from ketones, 4.6% from amino acids, 2.8% from lactate, and 2% from acetate [15]. In patients with heart failure (left ventricular ejection fraction < 40), ATP production from ketones was found to nearly tripled (16.4%) and that of lactate had nearly doubled (5.0%) [15]. Circulating ketone body concentrations were increased in patients with heart failure, in correlation with cardiac use. The concentrations of βOHB in circulation were also increased in persons with incident heart failure [115]. Circulating levels of ketone bodies and their myocardial use were also found increased in persons with type 2 diabetes [14].

One reason for increased ketone body usage in the diabetic or failing heart may be that ketolysis yields more energy available to synthesize ATP than fatty acid oxidation [116]. However, acute increases of ambient ketone bodies in the perfused mouse heart did not improve cardiac work, although at 2 mmol/l βOHB, ketolysis became the major source of energy production and TCA cycle activity and oxygen consumption were markedly increased [117].

This indicates that, as discussed before, beneficial effects of ketone bodies in the heart require an adaptive cellular response. For one, there is enzyme adaptation to promote βOHB breakdown and a longer myocardial transit time allowing better extraction from the blood [15, 118, 119]. Second, pretreatment of rats for many weeks with a ketogenic diet or 3-day fasting conferred cardioprotective effects in ischemia-reperfusion experiments, compared with a control diet [120, 121]. High concentrations of racemic βOHB reduced myocardial infarct size of isolated rat hearts after coronary artery occlusion and reperfusion only if rats were fasted for several days prior to the experiment [122]. Treatment of mice with βOHB before and during the 24 h reperfusion period after 30 min of ischemia decreased mitochondrial ROS production, increased ATP formation, and improved further parameters of myocardial cell injury including endoplasmic reticulum stress [123].

**Fig. 3:**
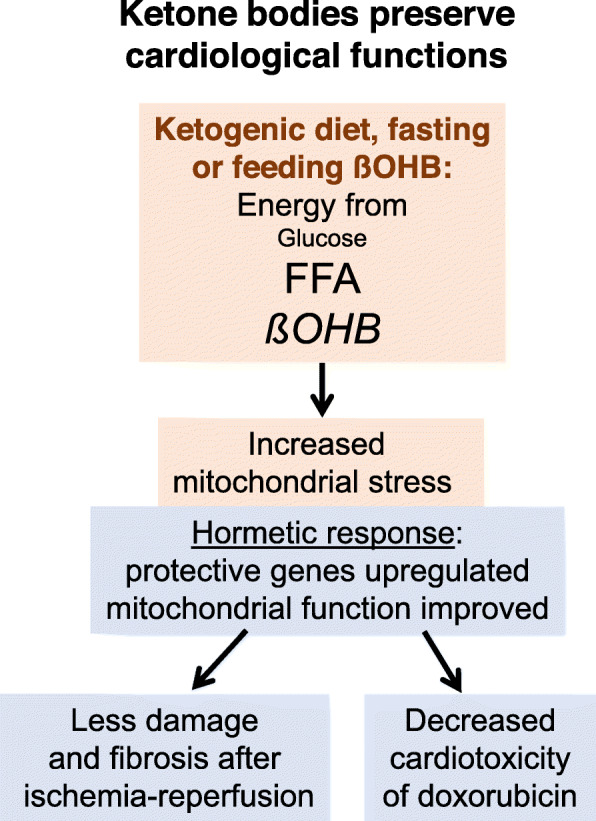
Ketone bodies preserve cardiological functions in animal studies. Increasing ketone body utilization by cardiomyocytes via fasting, ketogenic diet, or supplementing βOHB causes mitochondrial stress which is followed by an adaptive cellular response which is characterized by improved mitochondrial function and anti-oxidative defense. This leads to less cell damage and fibrosis in ischemia-reperfusion experiments and less cardiotoxic effects of doxorubicin. FFA, free fatty acids

The adaptive response protecting against myocardial ischemia-reperfusion injury apparently requires a priming phase characterized by increased circulating ketone bodies and their mitochondrial oxidation as an essential condition. Blocking ketolysis by suppressing gene expression of cardiac-specific BDH1 in adult mice eliminated the protective effect of high circulating ketone body levels [124]. This observation argues against a protective effect of βOHB as an intact molecule such as by blocking class I or II histone deacetylases but favors the hormetic pathway discussed above, i.e., metabolizing ketones at high rate causes moderate oxidative stress followed by an adaptive cell protective response including upregulation of anti-oxidative and anti-inflammatory gene products, and of mitochondrial function. Upregulation of these genes is known to mitigate the damage caused by myocardial ischemia and reperfusion [125, 126]. The chemotherapeutic drug doxorubicin causes cardiotoxicity via acute mitochondrial injury. Treatment of mice for 5 days with βOHB or a cardiomyocyte cell line for 24 h prevented doxorubicin-induced cardiac function decline and fibrosis in vivo, reduced oxidative stress, and maintained mitochondrial function in vitro [127]. Again, this fits with the concept of βOHB as an inductor of an adaptive (hormetic) cell defense response (Fig. 3).

### Increasing ketone body levels by pharmaceutical intervention

The blood levels of ketone bodies cannot only be increased despite the absence of a ketogenic diet by exogenous ketone body salts or esters but also by pharmacological treatment. Treatment of type 2 diabetes with sodium-glucose co-transporter 2 (SGLT2) inhibitors for decreasing elevated blood glucose concentrations by less reabsorption in the kidney was found to increase systemic ketone body levels. The ketonemia seen during the administration of SGLT2 inhibitors usually is around 1 mmol/l which corresponds to levels seen after 1–2 days of fasting. The lowering of blood glucose levels was accompanied by a decrease in circulating insulin concentrations, increased glucagon levels, and gluconeogenesis. The resulting increase of lipolysis and shift to enhanced usage of fatty acids for energy production promotes ketogenesis [128, 129]. Concomitantly, the risk of cardiovascular events was reduced. Treatment of type 2 diabetes with the SGLT2 inhibitor empagliflozin led to a reduction of cardiovascular mortality by 38% and of hospitalizations because of heart failure by 35% [130], similar to cardiovascular outcome trials with dapagliflozin and canagliflozin [131, 132]. The treatment curves SGLT2 inhibitor vs placebo began to diverge within 1 month [133]. These benefits cannot be explained solely by an action of SGLT2 inhibitors to lower blood glucose because similar effects are not seen with glucose-lowering drugs that have a stronger effect on glucose decrease, such as insulin, and because SGLT2 inhibitors also work in patients without diabetes and improve heart failure [134]. Likewise, lowering blood pressure does not appear to be involved because cardioprotection by SGLT2 inhibition is seen in patients receiving additionally other more potent antihypertensive medication [131–134]. The cardioprotective effects cannot be ascribed to a natriuretic action, since these SGLT2 inhibitors exert only a modest effect on plasma volume or on circulating natriuretic peptides [130, 135].

**Fig. 4:**
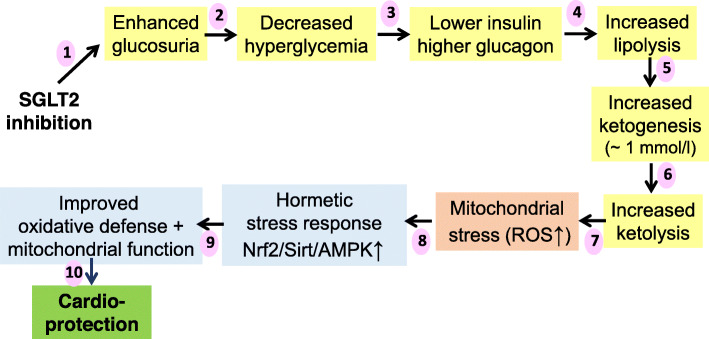
Suggested mechanism for the cardioprotective action of SGLT2 inhibitors. The lowering of blood glucose levels because of suppressed reabsorption in the kidney leads to lower systemic insulin and higher glucagon levels and resurgence of lipolysis resulting in substantial ketogenesis. Increased ketolysis in the heart causes mitochondrial stress followed by a protective (hormetic) response leading to improved mitochondrial function and anti-oxidative capacity which provides significant cardioprotection. SGLT, sodium-glucose co-transporter. (1), (2), (3), see references [129, 155]; (4) and (5), see references [128, 129]; (6), see references [14, 136]; (7), see references [18–25, 156]; (8) and (9), see references [29, 31–34]; and (10), see references [123–127]

Therefore, it has been proposed that the beneficial effects of ketone bodies on cardiac function account for the cardioprotective action of SGLT2 inhibitors [136]. We wish to modify this hypothesis by suggesting that the major mechanism of cardioprotection is not the provision of readily available energy by βOHB [137] but that the key contribution is the hormetic action of ketone bodies causing a cell-protective phenotype of cardiomyocytes, endothelial cells, and other cell types of the heart. This concept fits with the observation that SGLT2 inhibitors promote anti-oxidative defense mechanisms, exert anti-inflammatory actions, mitigate fibrosis, or other cardiac remodeling [138, 139]. As described above, the hormetic actions of ketone bodies are mediated via ketone stress-induced Nrf2, AMPK, and sirtuins, all of which increase the production and/or activity of enzymes involved in ROS neutralization, detoxification, DNA repair, proper protein folding during endoplasmic reticulum stress, autophagy, and regeneration. In parallel, pro-inflammatory mediators are downregulated. Support for this concept comes from reports that treatment with SGLT2 inhibitors induce the activity of Nrf2 [138–140], of AMPK [139–146], and of sirtuins [135, 145, 147], accompanied by downregulation of the inflammasome NLRP3 [144, 148, 149] and prevention of doxorubicin cardiotoxicity [150]. Hence, SGLT2 inhibitors induce the same spectrum of cardioprotective mechanisms as seen for treatment with exogenous ketone bodies (Fig. 4).

Chronic kidney disease is another clinical situation where treatment with SGLT2 inhibitors is of benefit [151]. Whether the hormetic mechanisms described above contribute to the observed reduction of intraglomerular pressure is not known.

### Risks of ketoacidosis

A theoretical risk of nutritional ketosis or supplementing ketone body salts or esters is ketoacidosis. In such a situation, the concentrations of ketone bodies may reach 25 mmol/l [152]. These levels overpower the capacity of the body to buffer ketotic acids and keep the blood at a pH of around 7.4, in the context of severe hyperglycemia and glucosuria plus concomitant renal loss of sodium and potassium required for buffering ketotic acids. The acidic milieu in the blood may reach a pH of below 7.30 or even below 7.10. The association of enhanced ketone body production and decreased buffering capacity of blood may also lead to ketoacidosis in patients with type 2 diabetes or even in the absence of hyperglycemia, such as during alcohol abuse or treatment with SGLT2 inhibitors [153, 154].

In metabolic healthy persons, systemic concentrations of ketone bodies may reach 5–7 mmol/l, after prolonged fasting, or up to 5 mmol/l during a ketogenic diet (Table 1). However, the buffering power available during a healthy metabolic state is able to cope with such amounts of ketotic acids and a normal pH of the blood is maintained. Higher levels of circulating ketone bodies do not occur during starvation because fasting blood insulin levels remain in the low normal range which is sufficient to prevent an unrestricted increase of lipolysis. Partial inhibition of lipolysis and stimulation of some lipogenesis is a property of insulin already seen in the normal range of blood insulin levels [1].

### Conclusions (Table 2)

Energy metabolism of the liver is fine-tuned in such a way that the synthesis and secretion of ketone bodies are increased substantially during fasting, starvation or low availability of dietary carbohydrates, and the resulting lower insulin levels. Two special features of liver metabolism are of relevance. For one, hepatocytes engage in gluconeogenesis if the supply of dietary carbohydrates is scarce. Glucose synthesis uses up most of the available oxaloacetate and its precursors so that only little acetyl-CoA resulting from beta oxidation of fatty acids can be channeled into the TCA cycle. Rather, acetyl-CoA is converted into ketone bodies. A second feature promoting hepatic ketone body production and secretion is the lack of the enzyme SCOT which is essential for ketolysis. Ketogenesis thus is a one-way pathway in hepatocytes. Therefore, an unavoidable consequence of food shortage is a tenfold or higher rise of ketone body levels in circulation. We propose here that the ketone body represents a danger signal of impending energy loss and a guardian angel which prepares the body to cope with this situation, by induction of a cell-protective hormetic response.

**Table 2:** Key messages

• Starvation or very low availability of digestible carbohydrates lowers insulin levels and alters hepatic energy metabolism in favor of fatty acid-derived ketone body production and gluconeogenesis.	
• Ketone bodies β-hydroxybutyrate and acetoacetate can be used as energy substrates in most cell types of the body via ketolysis in the mitochondria.	
• The increased concentration of β-hydroxybutyrate appears to function as a danger signal by leading to improved resistance to oxidative and inflammatory stress and reduced total energy expenditure.	
• We propose that most protective effects of beta-hydroxybutyrate are not mediated by acting as a ligand for cellular targets but by increased ketolysis which initially causes moderate mitochondrial stress.	
• Ketolysis-induced mitochondrial stress that initiates an adaptive (hormetic) response is characterized by the activation of the master regulators of cell-protective mechanisms, Nrf2, sirtuin 1,3, and AMPK.	
• The hormetic response includes upregulation of genes and mediators involved in anti-oxidative and anti-inflammatory activities, improved function and growth of mitochondria, DNA repair, and autophagy.	
• Probably, all organs of the body may benefit from these actions. Best researched is the heart where increased ketolysis decreases damage after ischemia-reperfusion or after exposure to cardiotoxic doxorubicin.	
• The cardioprotective action of SGLT2 inhibitors is well explained by the upregulation of ketone body levels and the subsequent cell-protective hormetic response.	
• High insulin levels suppress lipolysis-dependent ketogenesis and the associated beneficial hormetic responses.	

It is well known that ketone bodies not only serve as ancillary fuel substituting for glucose in most cell types but also induce several other physiological responses which include anti-oxidative, anti-inflammatory, and cardioprotective features. The prevailing ketone body βOHB binds to several target proteins including histone decarbolases, histones, or G protein-coupled receptors. However, it is not known whether these properties relate to the observed beneficial effects of βOHB. We propose here that ketolysis itself is the major mechanism of protective ketone body function. Increased ketolysis causes oxidative stress in the mitochondria which in turn causes a cellular adaptive (hormetic) response characterized by the activation of the master regulators of cell-protective mechanisms, Nrf2, sirtuins 1 and 3, and AMPK. This hormetic response includes upregulation of genes and mediators involved in anti-oxidative and anti-inflammatory activities, improved function and growth of mitochondria, DNA repair, autophagy, and decreased energy expenditure for anabolic purposes.

Probably, all organs of the body may benefit from these actions. In the heart, hormesis following increased ketolysis decreases damage and fibrosis after ischemia-reperfusion or after exposure to cardiotoxic doxorubicin. Although there is no direct evidence, it seems probable that the cardioprotective action of SGLT2 inhibitors involves the same cell-protective hormetic response in the mitochondria stressed by increased ketolysis. Experimental and clinical data support the suggested pathway of SGLT2 inhibitor action, involving a decrease of blood glucose levels via lower insulin and higher glucagon levels, increased lipolysis and gluconeogenesis, enhanced ketogenesis in the liver, and ketolysis in the heart causing mitochondrial stress, followed by an adaptive response causing upregulation of anti-oxidative and anti-inflammatory capacities as well as improved mitochondrial function, finally resulting in improved cardiovascular functions and resistance to ischemic insults. In this context, low systemic insulin levels are essential. High insulin concentrations in the blood prevent the breakdown of endogenous fat stores and thus suppress ketogenesis and the associated beneficial hormetic responses.

## Data Availability

Data for this review were identified by searches of MEDLINE, PubMed, and references from relevant articles using the search terms “ketone bodies,” “β-hydroxbutyric acid,” “ketogenic diet,” “SGLT2 inhibitors,” and “mitochondrial function and hormesis.” In order to limit the number of references, more recently published papers referring to several previously published articles were cited, if possible. Only articles published in English were selected.

## Abbreviations

AMP: Adenosine monophosphate
AMPK: AMP-activated protein kinase
ATP: Adenosine triphosphate
BDH: βOHB dehydrogenase
CCL2: C-C motif chemokine ligand 2
CO_2_: Carbon dioxide
CoA: Coenzyme A
CS: Citrate synthase
DNA: Deoxyribonucleic acid
FFA: Free fatty acids
FOXO: Forkhead box protein O
GPRs: G protein-coupled receptors
HbA1c: Gemoglobin A1c
HCA: Hydroxycarboxylic acid receptor
HDAC: Histone deacetylase
HDL: High-density lipoprotein
HMG: Hydroxy methylglutaryl
HMGCL: HMG-CoA lyase
HMGCS2: Hydroxy methylglutaryl-CoA synthase
HOMA-IR: Homeostasis model assessment of insulin resistance
IFN: Interferon
Il: Interleukin
KEAP: Kelch-like ECH-associated protein
LDL: Low-density lipoprotein
MCT1: Monocarboxylate transporter
NAD: Nicotinamide adenine dinucleotide
NADP: Nicotinamide adenine dinucleotide phosphate
NF-kB: Nuclear factor “kappa-light-chain-enhancer” of activated B cells
NLRP3: NOD-, LRR-, and pyrin domain-containing protein 3
NOX: NADPH oxidase
Nrf2: Nuclear factor erythroid 2–related factor 2
ROS: Reactive oxygen species
SCOT: Succinyl-CoA:3-oxoacid-CoA transferase
SGLT2: Sodium dependent glucose co-transporter 2
SIRT: Silent information regulator sirtuin
SKN: SKiNhead
TCA: Tricarboxylic acid
TNF: Tumor necrosis factor
βOHB: β-Hydroxybutyrate
